# Surface Treatment of Polyamide 6 through Enzymatic Hydrolysis and Covalent Incorporation of Chitosan Nanoparticles

**DOI:** 10.1021/acs.biomac.4c01281

**Published:** 2025-01-15

**Authors:** Larissa Paza, Wendhy C. Vicente, Marília Miotto, Marcel Afonso Provenzi, Daniane Aparecida Netzel, Larissa N. Carli, Patrícia B. Brondani

**Affiliations:** † Centro Tecnológico, de Ciências Exatas e Educação, Federal University of Santa Catarina, João Pessoa Street, 89036-002 Blumenau, Brazil; ‡ Departamento de Ciência e Tecnologia de Alimentos, Federal University of Santa Catarina, Admar Gonzaga Street, 88034-001 Florianópolis, Brazil; § Departamento de Química, Federal University of Santa Catarina, Roberto Sampaio Gonzaga Street, 88040-380 Florianópolis, Brazil

## Abstract

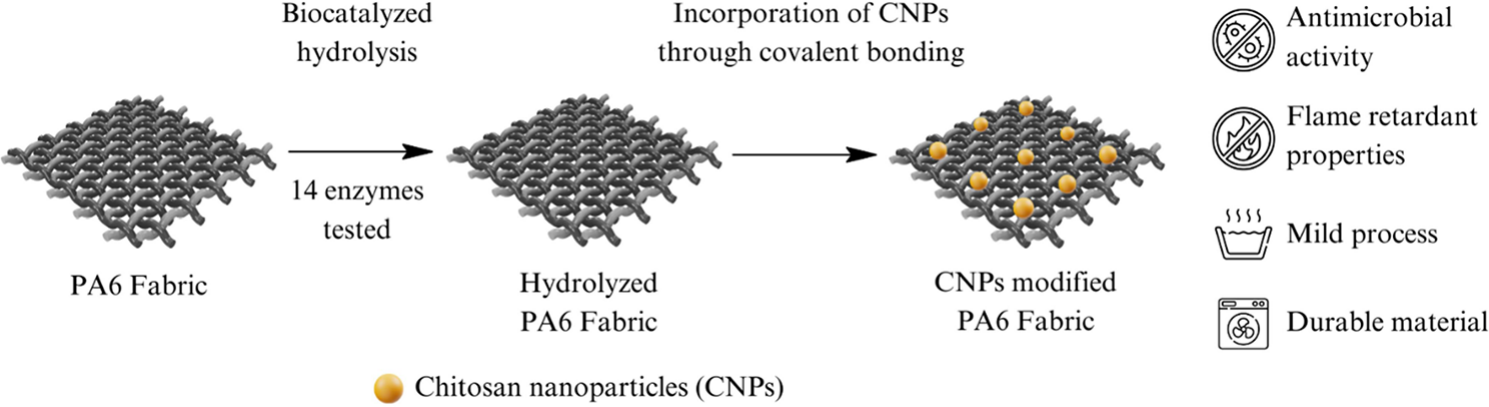

Polyamide (PA) has notable physical and chemical properties and is one of the most versatile synthetic materials in the industrial sector. However, its hydrophobicity creates significant challenges in its beneficiation and modification. Modifications of PA with chitosan nanoparticles (CNPs) can improve its undesired properties but are rarely found in the literature due to the weak interaction between the chemical groups of both structures. Surface hydrolysis mediated by enzymes can mildly improve the PA properties and create reactive sites. These sites can react with CNPs to confer enhanced properties to the fabrics, such as antimicrobial activity and flame retardancy. This study investigated the action of 14 hydrolases in the surface hydrolysis of 100% polyamide 6 (PA 6) fabric. Such an extensive study applying several enzymes for this process is uncommon. Under the optimum conditions, the hydrolyzed fabric was covalently bonded to the CNPs, generating material with reduced bacterial proliferation and flame retardancy properties. The uncommon covalent bond attachment achieved high material durability, even after five washing cycles.

## Introduction

Polyamide (PA) fabric has physicochemical properties that make it one of the most versatile synthetic materials in the industry. Composed of polyethylene (CH_2_)_n_ segments separated by peptide units, its properties of elasticity, high mechanical strength, lightness, and abrasion resistance allow it to be used in countless applications in the textile industry, such as the manufacture of sportswear, disposable clothing for the health sector, hosiery, lingerie, sewing threads, carpets, safety belts, and more.^1,2^

However, like other synthetic fabrics, PA has low moisture absorption properties, inefficient water retention, and a propensity to retain static charges. The inherent hydrophobicity of these materials imposes significant challenges during the beneficiation process, such as dye penetration difficulties during the dyeing process, adhesion of coatings, static effects, breakages, problems associated with washing, and costs of the process.^3^ These drawbacks can be partially overcome by surface modification through fabric functionalization.^4−6^ Conventional functionalization methods described in the literature require aggressive chemical agents and high energy consumption, which include acid and alkaline hydrolysis, generating effluents, a significant environmental impact, and damage to the fabric structure.^7−10^ However, milder methodologies that maintain the quality of the textile substrate can be achieved using biocatalysis.

Hydrolases are enzymes that catalyze reactions involving the hydrolytic rupture of chemical bonds. Therefore, the use of hydrolases such as lipases, proteases, papain, and trypsin in the treatment of PA textile substrates results in the hydrolysis of the fabric’s amide groups, leading to the formation of carboxyl and amine groups, which increases hydrophilicity, improving some stages of the beneficiation process, among them the dyeing properties.^1,6,11−14^ Kanelli et al., for example, hydrolyzed the surface of polyamide 6.6 with the protease enzyme, increasing the hydrophilicity of the fabric by 2.7 times, as evidenced by a 1.24 times increase in dye yield, with no impact on the original properties of the substrate.^15^

Several studies apply enzymes to hydrolyze the surface of polyamide, but few study a wide range of enzymes in complete optimization studies. These studies are essential because enzymatic treatment reduces the amount of water and energy used during the process, being milder and minimizing the use of chemical products that can harm the fabric and the environment when discarded.^16−18^ Furthermore, enzymes can be used simply and in equipment already available within the textile industry.^19−21^

In addition to the previously mentioned improvement in hydrophilicity with minimal damage to the fabric, enzymatic hydrolysis generates reactive groups on the fabric’s surface, which can subsequently be functionalized. The COOH groups generated can react with nucleophilic species in further modifications, developing a covalent bond. Reacting these groups with other molecules is a promising way to give new properties to fabrics.^21−24^

These properties can be altered, for example, by bonding nanoparticulate materials. With advances in nanotechnology, various nanoparticles, such as polymeric nanoparticles or nanocomposites, have been effectively incorporated into synthetic fabrics, resulting in functionalized fabrics with differentiated properties.^25,26^ The application of nanotechnology in functionalizing textile substrates results in improved processes and the development of fabrics with new properties without harming their original properties of lightness, comfort, flexibility, and durability, thus adding additional value to the product.^27−29^

Among the nanostructured materials used to develop functional textiles, chitosan nanoparticles (CNPs) are an exciting alternative that can confer antimicrobial, antifungal, flame retardant, and ultraviolet (UV) protection properties, in addition to being biodegradable and nontoxic.^30−38^ Few studies have focused on the covalent bonding of nanoparticles to the surface of textile substrates, even though this bonding can confer high durability to the material generated.^34^ When discussing CNPs and synthetic fabrics, the simple incorporation, even by intermolecular bonds, is complex and less usual due to the chemical nature of the fabrics.^30−38^

This work aimed to study the action of various enzymes in the surface hydrolysis process of 100% polyamide 6 (PA 6) fabric and to impregnate chitosan nanoparticles (CNPs) to the fabric by covalent bonding. The hydrolysis reaction conditions were fully optimized by studying the type of enzyme used and its concentration, temperature, reaction time, and pH of the medium. The hydrolysis efficiency was evaluated by dyeing the fabric with a basic dye. After the optimization, the covalent incorporation of the CNPs was performed, and the characteristics of the material generated were evaluated, as well as the multiple functionalities conferred by chitosan, such as antimicrobial action and flame retardant, and the washing resistance.

## Experimental Section

### Materials

The study of enzymatic treatment on PA 6 was carried out using 100% polyamide 6 with an interlock structure donated by Texneo. The nonionic surfactant ethoxylated alcohol with C12–C16 90% (Berol 175), donated by Macler, was used to purge the substrate.

The enzymatic modification was carried out with 14 different commercial enzymes obtained from Sigma-Aldrich, as shown in Table 1. All the enzymes were used as received and stored at −20 °C.

**Table 1:** Code of the Hydrolyzed Samples and Respective Enzymes Used in the Enzymatic Treatment of PA6

code	enzyme description
*PAHidCR*	Lipase from *Candida rugosa* type VII (lyophilized)
*PAHidBCi*	Lipase PS (Amano) from *Burkholderia cepacia* (immobilized in silica)
*PAHidBC*	Lipase PS (Amano) from *Burkholderia cepacia* (lyophilized, PS-SD)
*PAHidRO*	Lipase F-AP15 (Amano) from *Rhizopus oryzae* (lyophilized)
*PAHidRM*	Lipase from *Rhizomucor miehei* (in solution, Novozyme 388)
*PAHidPP*	Lipase from *Porcine pancreas* type II (lyophilized)
*PAHidAN*	Lipase A (Amano) from *Aspergillus niger* (lyophilized)
*PAHidTL*	Lipase from *Thermomyces lanuginosus* (lyophilized)
*PAHidRM*	Lipase from *Rhizomucor miehei* (immobilized in ion-exchange resin type Duolite, Lipozyme RM-IM)
*PAHidMJ*	Lipase M (Amano) from *Mucor javanicus* (lyophilized)
*PAHidTLs*	Lipase from *Thermomyces lanuginosus* (immobilized in sol–gel)
*PAHidPA*	*Papain*
*PAHidTRI*	*Trypsin*
*PAHidPR*	*Protease A*

The enzymatic treatment was carried out using a 0.05 mol L^–1^ Tris(hydroxymethyl)aminomethane buffer solution, pH 6.0, 7.0, and 8.0, prepared using a commercial Vetec reagent and pH adjusted with hydrochloric acid (HCl) or sodium hydroxide (NaOH). The basic and acid dyes used were Methylene Blue (CI 52015) obtained from Neon and Bemaplex Red MT (CI Acid Red 315) by CHT. The acid donor Meropan EF was used to perform the acid dyeing. The detergent Ekotex Clean performed the washing cycles and fastness resistance.

The nanoparticles were synthesized using low-molar mass chitosan from Sigma-Aldrich (75% deacetylation). The acetate buffer solution 0.1 mol L^–1^, pH 5.0, was prepared with sodium acetate (CH_3_COONa) (Dinâmica) and acetic acid (CH_3_COOH) (Lafan). The 0.1% TPP solution was prepared in distilled water from a commercial reagent (Dinâmica).

The nanoparticles’ enzymatic treatment, dyeing, and incorporation were carried out in an orbital shaker/incubator from SOLAB, model SHAKER SL-122. A Shimadzu 1800 UV–vis spectrophotometer using UVProbe software (version 2.42) and a glass cuvette with a 1 cm optical path were used to analyze the dye bath. The color strength (K/S) of the dyed samples was measured with a DataColor DC400 reflectance spectrophotometer using an ultrasmall slit of 6 mm under a D65 illuminant with a 10° standard observer (D65 10 Deg).

### Pretreatment of PA6

Impurities were removed from the fabric’s surface by washing it in a domestic washer-dryer (Electrolux) in a bath containing 2 mL L^–1^ of nonionic detergent (ethoxylated alcohol), using a delicate wash program at 50 °C for 60 min at gentle spinning.^1^ The fabric was dried at room temperature and stored in a dry place.

### Synthesis of Chitosan Nanoparticles

The CNPs were synthesized through the ionic gelation method. A chitosan solution (1% w/v) in acetate buffer 0.1 mol L^–1^ pH 5.0 was prepared. The solution was kept under stirring for 1 h. After 24 h, it was centrifuged at 2500 rpm for 30 min and filtered under vacuum. The solution was mixed with a 0.1% sodium tripolyphosphate (TPP) in a 3:1 ratio of chitosan:TPP (v/v) and kept under agitation for 1 h at 25 °C. It was then stirred in an IKA Ultra-Turrax disperser (T 18 DI with S 18 N dispersion element) at 13,000 rpm in an ice bath for 10 min. The CNPs obtained were kept in a refrigerated solution at 4 °C.^35^

### Characterization of Chitosan Nanoparticles

A JEM-1011 transmission electron microscope (TEM) was used with 80 kV of acceleration tension, with images at magnifications of 10,000× to 100,000× for the morphological evaluation of the synthesized nanoparticles. Subsequently, the images were processed using the ImageJ software to analyze the diameter of the nanoparticles. The nanoparticles were previously dispersed in distilled water and sonicated for 20 min. This dispersion was deposited on a 300-mesh copper grid covered with carbon film and dried for 48 h.

### Surface Hydrolysis of PA6 and Optimization of Reaction Conditions

The enzymatic modification was carried out with 14 different enzymes to identify the one with the best performance in the hydrolysis of PA 6. All the reactions were carried out in duplicate. Samples measuring approximately 2 cm × 2 cm were cut out, weighed, and identified according to the enzyme used. A total of 10% (w/w) of the enzyme was weighed, and 24 mL of 0.05 mol L^–1^ Tris-HCl buffer solution pH 7.0 was added to 50 mL beakers, with a liquor ratio of 80.^39^ The solution was incubated at 40 °C for 90 min, with 150 rpm agitation. Afterward, the samples were washed with distilled water for 10 s at 25 °C and dried at room temperature.

To optimize the reaction parameters, the enzyme treatment procedure was repeated with the best enzyme while varying the pH (6.0, 7.0, and 8.0), temperature (25, 40, and 60 °C), reaction time (45, 6,0, and 90 min) and enzyme concentration (5, 10 and 15%). The condition that led to the best hydrolysis results was pH 8.0, 40 °C, 90 min, and 5% lipase from *Porcine pancreas* type II (lyophilized).

### Verification of Surface Fabric Modification

PA 6 was dyed with methylene blue (MB) in an incubator under agitation at 150 rpm, with 0.04 g of fabric and 2.5% (w/w) of dye, at pH 7.0 and 30 °C for 30 min.^1^ The tests were duplicated, with one test with the fabric without enzymatic treatment for each step. The dyed samples were washed with distilled water for 10 min at 25 °C under 150 rpm agitation and dried at room temperature.

The exhaustion baths were reserved and subjected to absorbance measurements in the UV–vis spectrophotometer, using a calibration curve for the dye that correlates the concentration with the established absorbance. This method allows for determining the concentration of residual dye in the dye bath and the concentration of dye adsorbed by the sample.

The dyed samples were measured regarding reflectance on a DataColor spectrophotometer to analyze the colorimetric properties of intensity expressed by the dye strength value (*K*/*S*). The *K*/*S* was determined using the Kubelka–Munk equation, represented in eq 1.
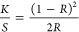
1where *R* is the reflectance, *K* is the dye’s absorption coefficient, and *S* is its dispersion coefficient. The wavelength with the lowest reflectance indicates the region where the dye absorbs the highest amount of light, serving as a valid indicator of color intensity.

### Incorporation of Chitosan Nanoparticles

The CNPs were incorporated into the PA 6 samples, which had been previously hydrolyzed by the selected lipase under optimized conditions, using the methodology developed by ALONSO et al.^40^ The process began with a preimpregnation, where the fabrics were immersed in a 20% (v/v) CNPs solution in acetate buffer (0.1 mol L^–1^, pH 5.0). The mixture was stirred at 150 rpm for 120 min at 60 °C. After this period, the samples were dried at room temperature. The preimpregnated samples were placed in beakers containing acetate buffer solution and subjected to ultraviolet radiation with 254 nm wavelength light for 240 min in a Dist GR 03 UV cabin. After irradiation, the impregnation process was repeated, following the washing and drying steps described above.

### Characterization of the Modified Fabric

To validate the effect of enzymatic hydrolysis and confirm the incorporation of the CNPs into the treated, the surface of the fabrics was evaluated in FTIR in the range 4500–400 cm^–1^ using 16 scans at a resolution of 4 cm^–1^ in a PerkinElmer Frontier spectrometer. The intensity of the band at 2933 cm^–1^, which refers to Csp^3^-H stretching, was used to normalize the spectra.

To evaluate the enzymatic treatment, the incorporation of the nanoparticles, and their durability, scanning electron microscopy (SEM) images were obtained using a ThermoFisher Scientific Prisma E scanning electron microscope, an acceleration of 10 kV, and a low vacuum mode of 50 Pa.

The contact angle was measured on a goniometer ramé-Hart 250 equipped with Drop Image software to evaluate the hydrophilicity of the fabrics. A drop of distilled water was directly deposited on the surface of each sample, measuring 1 cm × 3 cm. The test was carried out in triplicate.

Thermogravimetric analysis (TGA) was performed using PerkinElmer TGA8000 equipment to evaluate variations in the mass of the fabric samples concerning temperature, making it possible to assess the thermal stability of the modified fabric. The procedure was conducted under an argon atmosphere at a constant 20 mL/min flow rate. The heating rate applied was 20 °C min^–1^, from 30 to 700 °C.

Antimicrobial activity was evaluated using the standard method ASTM E2149–01, with modifications. Standardized inoculum of *Staphylococcus aureus* ATCC 25923 and *Escherichia coli* ATCC 25922—Gram-positive and Gram-negative microorganisms, respectively—were used. The inoculum was standardized in 0.9% NaCl saline using 0.5 McFarland standards. The samples (3 cm × 3 cm, 0.04 g) were mixed with 1 mL of the standardized inoculum, stirring at 200 rpm for 24 h at 36 ± 1 °C. After this contact time, serial decimal dilutions were performed, and plating was done on nutrient agar (Kasvi, Brazil). After 48 h of incubation at 36 ± 1 °C, counts were made, and the result was expressed in CFU/mL. Untreated material was used as a control and evaluated in the same conditions. The percentage reduction was calculated based on eq 2, comparing the functionalized fabric count with the control after 24 h of contact.^34,35^ The test was carried out in triplicate.
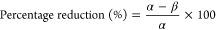
2where α and β are the number of bacteria recovered from the nonfunctionalized and functionalized fabrics, respectively, after 24 h of inoculation and incubation.

The vertical flame test, using the standard method ASTM D6413-08, was carried out to assess the flame-retardant properties of the modified fabric. An apparatus was constructed so that the samples of dimension 9.5 cm × 1.0 cm were hung up, and their ends were exposed to the flame for a standardized period of 2 s. The flame was ignited with a gas torch containing 3% propane, 63% nor-butane, and 34% iso-butane, set at minimum intensity. For all tests, there was 2.5 cm between the nozzle and the bottom edge of the sample.

The washing resistance was evaluated through five washing cycles on an impregnated sample to verify the durability of the functionalization of the fabric with the CNPs. Functionalized fabrics were washed according to standard ISO 105-C10. In each cycle, the fabric was immersed in a beaker containing 5 g L^–1^ detergent solution and stirred in an orbital shaker at 40 °C for 30 min at 100 rpm. After each cycle, the fabric was dried at room temperature before being subjected to the next washing cycle. The surface of the sample 5WC*PAHidPP_CNPs* was examined via SEM using the previously described conditions to analyze the CNPs’ permanence in the fabric after the washing cycles.

The dyeing properties of the modified fabric were assessed using the wash fastness test, adapting the methodology presented in ABNT NBR ISO 105: Color Fastness Tests—Part C06:2010: Color Fastness to Domestic and Commercial Washing. Unmodified PA 6, impregnated with CNPs and impregnated after five washing cycles (0.06 g), were dyed with Bemaplex Red MT acid dye (1.0% w/w) and Meropan EF acid donor (0.5% v/v). The dyeing of PA 6 fabrics was carried out using a high-temperature dyeing machine (Mathis ALT-ECO). The dyeing process started at 40 °C, with a raise to 95 °C over 20 min, then held the temperature for 60 min.^6^ The dyed samples were washed with distilled water for 10 s and dried at room temperature. These samples were placed in an incubator with 4 g L^–1^ of detergent and five stainless steel spheres with a diameter of 2 mm under agitation at 100 rpm for 30 min at 40 °C. The samples were then dried at room temperature and classified according to the grayscale, receiving scores from 1 to 5, where 1 indicates a high change in tone and 5 indicates no change in tone.

## Results and Discussion

### Synthesis of Chitosan Nanoparticles

**Figure 1:**
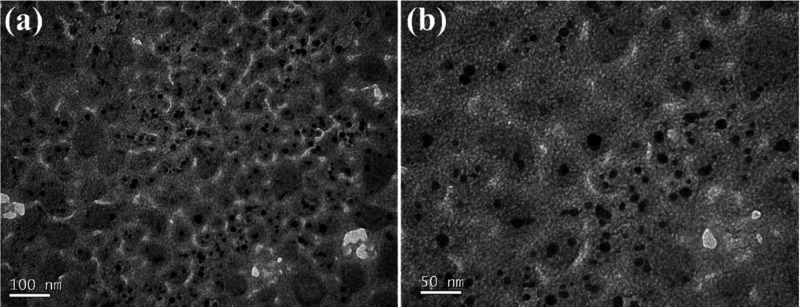
TEM micrographs of the synthesized chitosan nanoparticles on a scale of (a) 100 nm and (b) 50 nm.

The CNPs were synthesized using the ionic gelation method and the Ultra-Turrax disperser to reduce particle size. Transmission electron microscopy (TEM) images confirmed the formation of the nanoparticles (Figure 1a,b), which were spherical and uniformly dispersed, similar to some reports found in the literature.^41−43^ Raza et al. also prepared chitosan nanoparticles via the ionic gelation method and obtained spherical CNPs with an average particle size (hydrodynamic diameter) ranging from 115 to 540 nm. El-Naggar et al. synthesized CNPs using *Pelargonium graveolens* leaf extract with nonaggregation and lower-agglomeration status nanoparticles with similar morphology and particle size.^35^ This work’s size analysis revealed an average particle size of 10.60 ± 3.26 nm.^43^

### Surface Hydrolysis of PA6

**Scheme 1:**
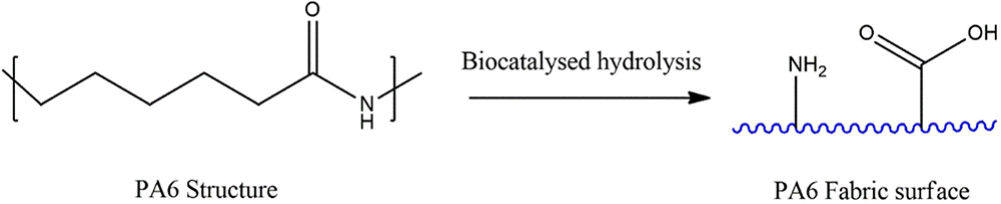
Enzymatic Hydrolysis of PA6

The first step in modifying the fabric was surface hydrolysis, in which various enzymes (10% w/w) were tested with the PA 6 fabric at pH 7.0, 40 °C for 90 min. The modified fabric was analyzed to determine which enzyme performed best. As shown in Scheme 1, the enzymes hydrolyze the amide bonds of the PA 6 fabric, generating carboxylic groups and amines on the fabric’s surface.

**Figure 2:**
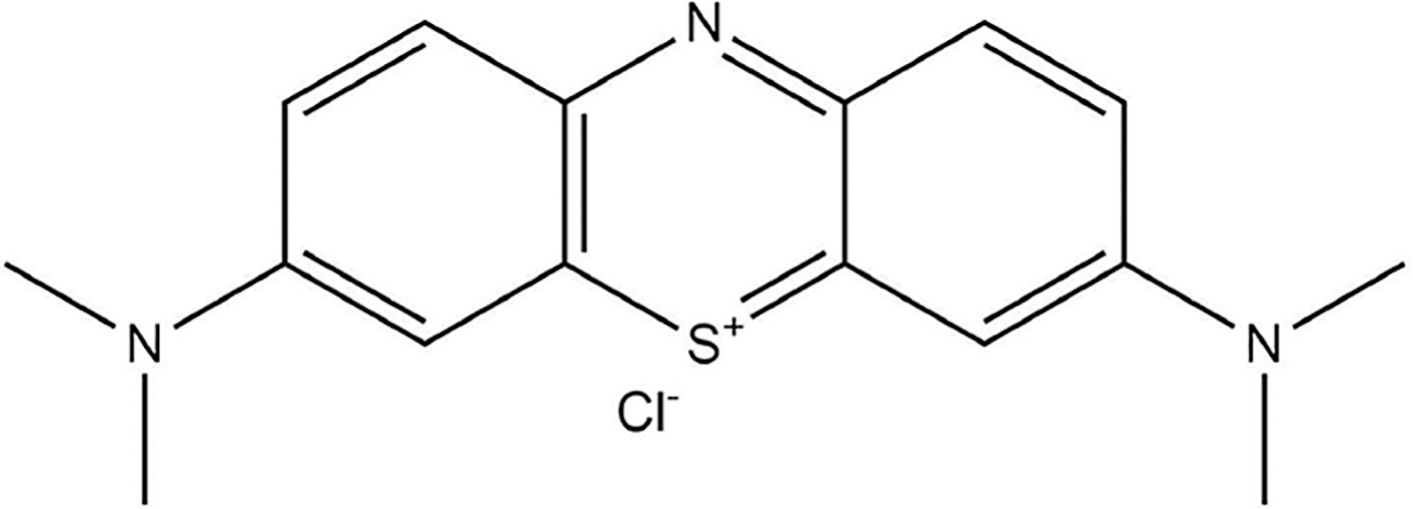
Methylene blue structure.

The presence of these groups on the surface of the pretreated fabric is an indicator of the efficiency of the hydrolysis of the polyamide and one of the ways to check and thus optimize the hydrolysis process. This presence can be verified through dyeing with MB, which the COO^–^ groups attract at the appropriate pH, as shown in Figure 2.^1,15^

After dyeing with the MB, the modified fabric shows higher *K*/*S* values than the unmodified one, which is visually represented by more intensely colored samples. Additionally, fabrics dyed more efficiently (more hydrolyzed) lead to a lower residual dye loss in the bath. Table 2 shows the quantification of the dye concentration in the samples.

**Table 2:**
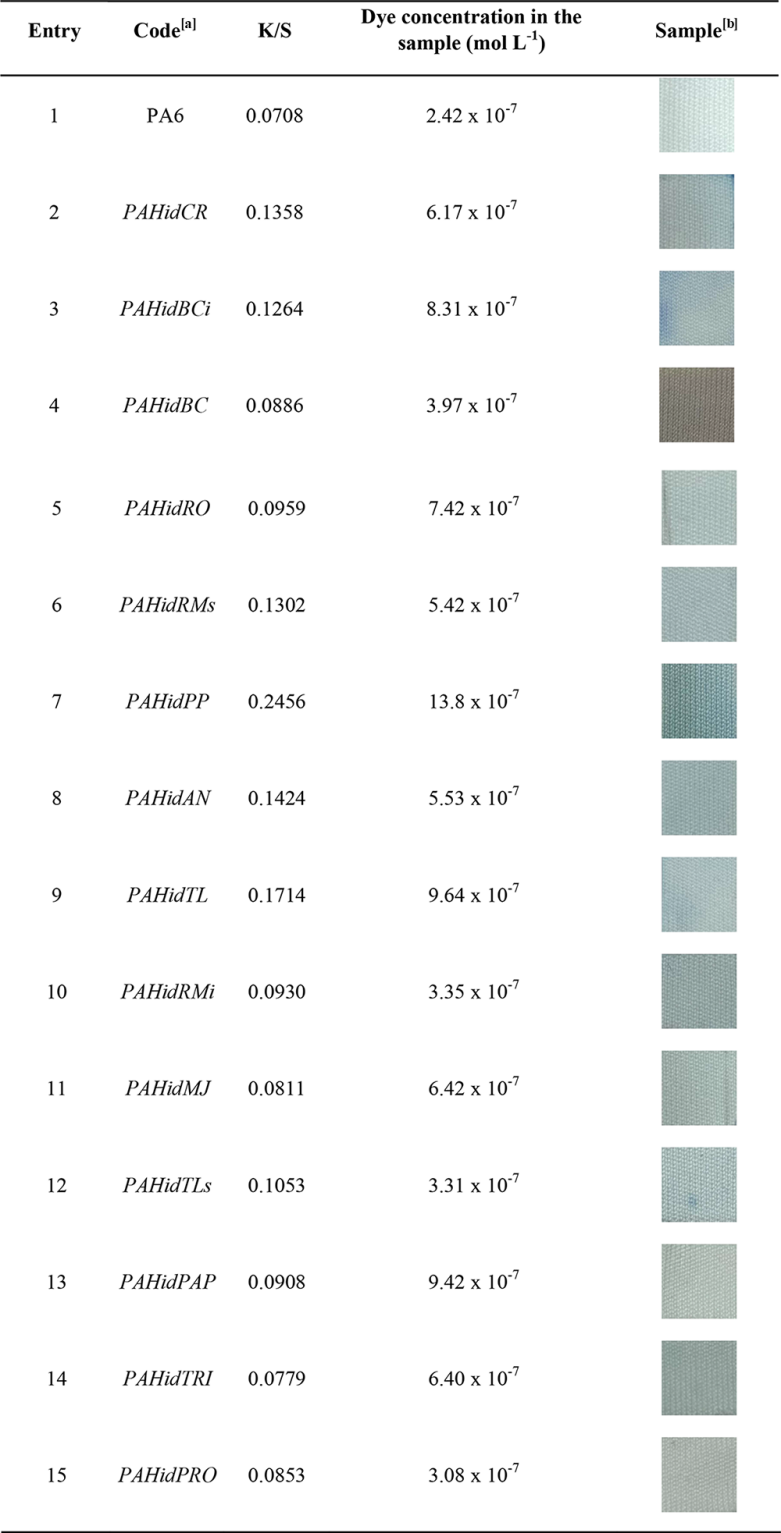
Dyeing with MB Analysis of Enzymatically Modified Samples, Represented by *K*/*S* Values and Dye Concentration in the Sample

a Treatment conditions: 10% of the enzyme (w/w), pH 7.0, 90 min, 40 °C.

b Dyeing conditions: 2.5% dye (w/w), pH 7.0, 30 °C, 30 min.

The lipase from *Porcine pancreas* type II (lyophilized) (*PAHidPP*) showed the highest hydrolysis efficiency, as seen in Table 2, entry 7. Applying this enzyme led to a modified fabric that generated the best dye yield, represented by the *K*/*S* value and dye concentration in the sample, which were 3.5 and 5.7 times higher than those of the unmodified fabric, respectively.

**Figure 3:**
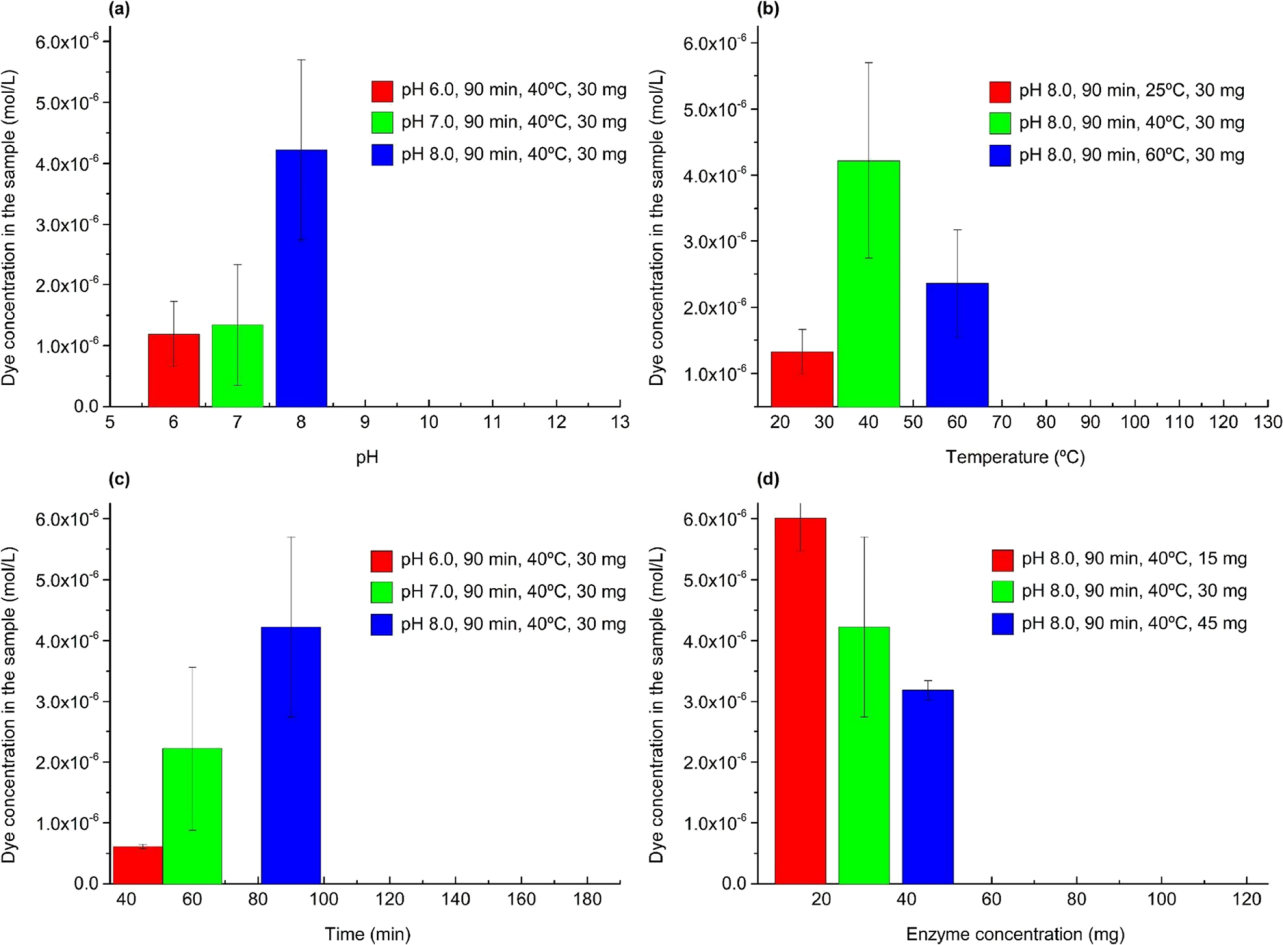
Concentration of MB after dyeing in *PAHidPP* samples is used to optimize (a) pH, (b) temperature, (c) reaction time, and (d) amount of enzyme.

After selecting the lipase with the best performance, the hydrolysis parameters were optimized by adjusting the pH (6, 7, and 8), temperature (25, 40, and 60 °C), reaction time (45, 60, and 90 min) and enzyme concentration (5, 10, and 15%). Varying these conditions showed a significant and quantifiable impact on the effectiveness of the enzymatic process, as evidenced by the adsorption of the dye under the different hydrolysis conditions. This relation is illustrated by the response to dyeing with MB, as shown in Figure 3.

Figure 3a shows that as the pH of the medium is raised, there is an increase in hydrolysis efficiency, observed through the progressive increase in the ability of the treated fabric to adsorb the dye, with the highest efficiency being achieved at pH 8.0, a value that coincides with the optimum pH documented for the selected lipase.^19,44^ Similarly, the influence of temperature on the process was equally significant. As illustrated by Figure 3b, the most efficient hydrolysis was found at 40 °C, a temperature in line with the optimum temperature for the selected lipase activity.^19,44,45^ Optimization of the reaction parameters revealed that a reaction time of 90 min and an enzyme concentration of 5% are the most effective conditions for hydrolysis, as shown in Figure 3c,d, respectively.

Under these conditions, the sample dyed with the MB dye showed *K*/*S* values and dye concentrations 5.9 and 4.4 times higher, respectively, compared to the fabric hydrolyzed under the initial conditions (Table 3). Additionally, these values were 20.5 and 24.8 times higher, respectively, compared to the unmodified fabric (Table 3).

**Table 3:**
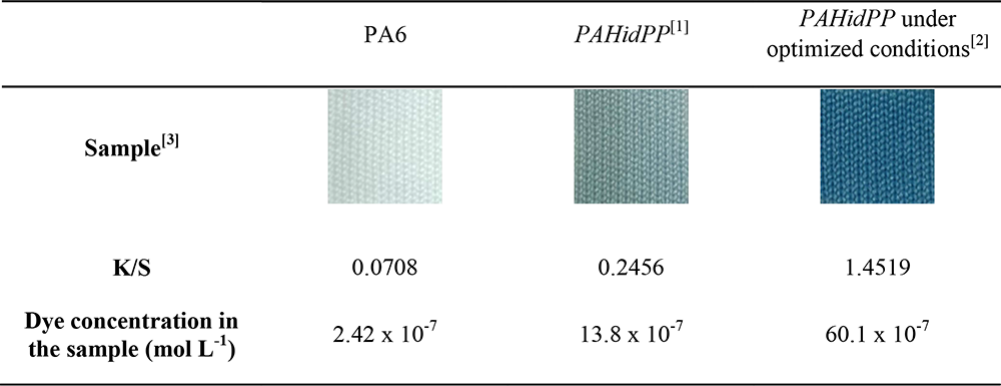
Visual Results, *K*/*S* Values, and Dye Concentration in the Sample for MB Dyeing in PA6 without Modification, *PAHidPP* in the Initial Conditions, and *PAHidPP* in the Optimized Conditions

a Treatment conditions: 10% (w/w) of the enzyme, pH 7.0, 90 min, 40 °C.

b Treatment conditions: 5% (w/w) of the enzyme, pH 8.0, 90 min, 40 °C.

c Dyeing conditions: 2.5% dye (w/w), pH 7.0, 30 °C, 30 min.

### Modification of the Fabric with Chitosan Nanoparticles

**Scheme 2:**
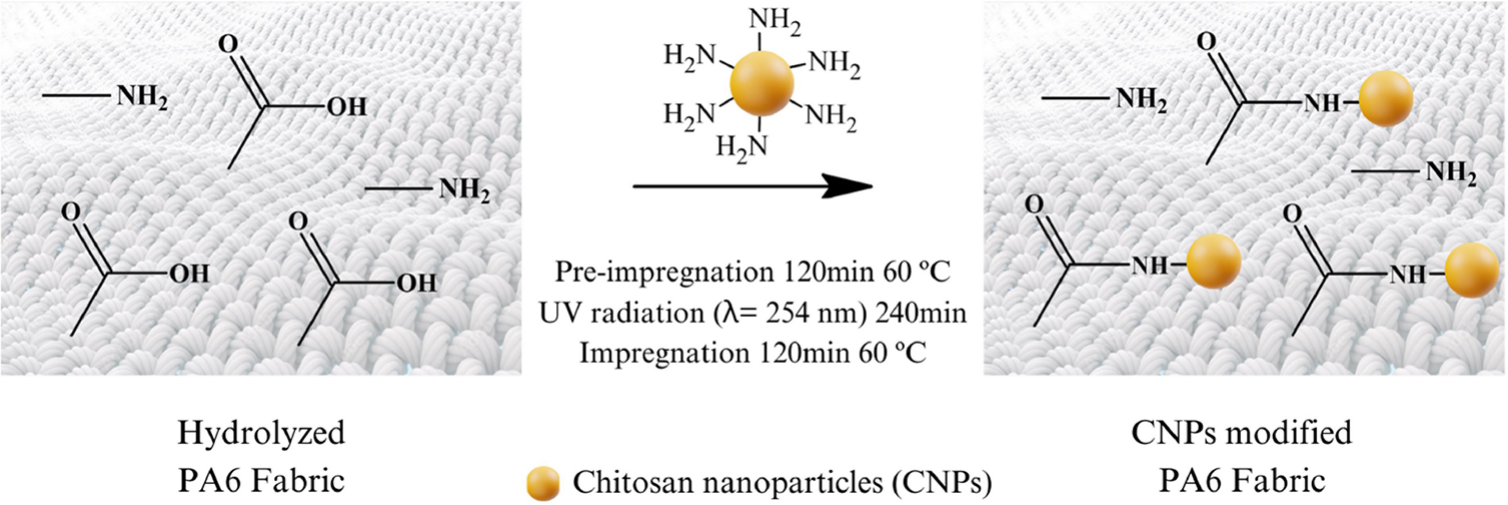
Surface Modification Process of PA6 Using Chitosan Nanoparticles

CNPs were incorporated into PA 6 samples, which were first hydrolyzed using the selected lipase under optimized conditions, as illustrated in Scheme 2. The purpose of the UV exposure was to enhance the incorporation of CNPs by increasing photo-oxidative degradation. This process generates free radical species in the nitrogen and oxygen atoms of the chitosan, making them available for coupling to the structures of interest, thereby improving the incorporation efficiency.^40^

**Figure 4:**
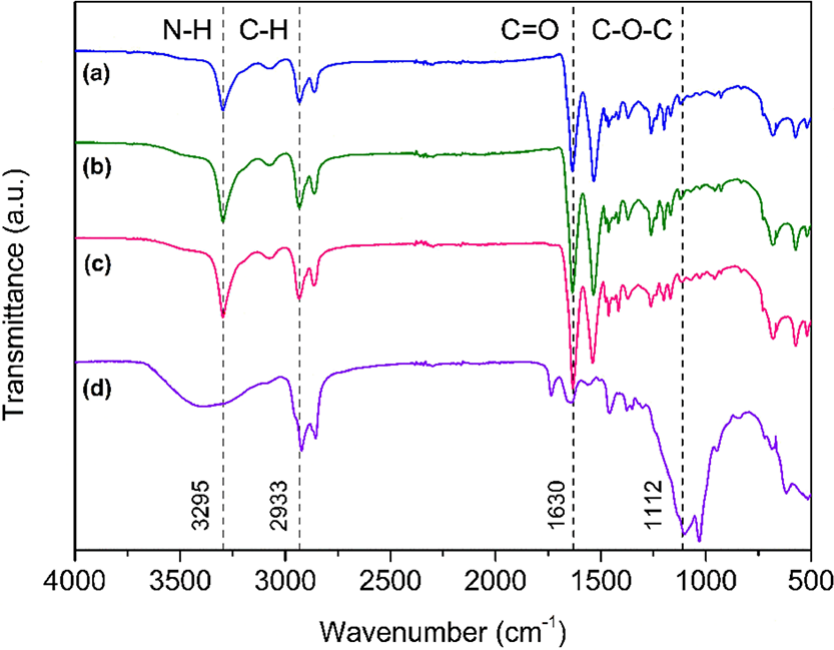
FTIR spectra for (a) PA 6 without modifications, (b) *PAHidPP* under optimized conditions, (c) *PAHidPP_CNPs,* and (d) CNPs.

FTIR was used to examine the structural changes in the PA 6 fabric. Figure 4 shows the FTIR spectra of polyamide in unmodified, hydrolyzed, and chitosan nanoparticle-impregnated conditions and the spectrum of the chitosan nanoparticles themselves.

The spectrum of unmodified polyamide (Figure 4a) shows characteristic bands of PA 6, for example, at 3295 and 2933 cm^–1^, which correspond to the stretching of the N–H and C–H bonds, respectively. The band at 1533 cm^–1^ represents the bending of the N–H bond, and the band at 1630 cm^–1^ is associated with C=O stretching, typical of the amide group.^35,46^

After enzymatic hydrolysis (Figure 4b), the spectrum shows an increase in the intensities of the bands at 3295 and 1630 cm^–1^, indicating the formation of N–H and COOH groups on the surface. This increase suggests that enzymatic hydrolysis was effective in modifying the chemical structure of the PA 6 fabric and can only be assessed by normalization.

In the chitosan nanoparticles spectrum (Figure 4d), characteristic bands were observed, such as at 3300 cm^–1^, corresponding to the N–H and O–H stretches. The C=O stretching of the carbonyl is observed around 1635 cm^–1^, and the bands between 1010 and 1150 cm^–1^ correspond to the C–O and C–O–C stretching vibrations.^46^

In the CNP-treated fabric, the intensities of the N–H, O–H, and C=O bands increase at 3295 and 1630 cm^–1^ (Figure 4c). In addition, the band’s intensity decreased at 1112 cm^–1^, which is related to the 1010–1150 cm^–1^ range characteristic of chitosan.^35,46^ These results confirm the presence of the bands typical of polyamide and chitosan nanoparticles, demonstrating that the treated fabric effectively incorporated the CNPs.

**Figure 5:**
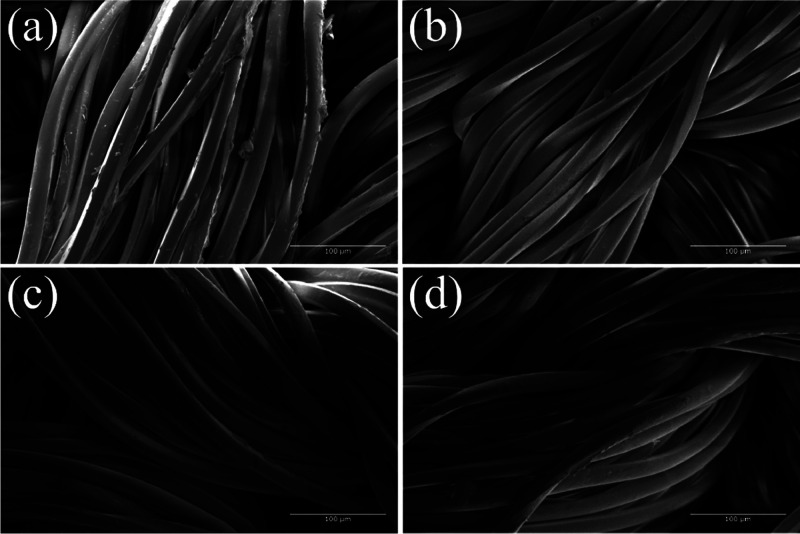
SEM images of (a) PA 6 without modifications, (b) *PAHidPP* under optimized conditions, (c) *PAHidPP_CNPs*, (d) 5WC*PAHidPP_CNPs.*

SEM images examined the surfaces of treated and untreated samples. Five washing cycles were also performed on a sample to investigate the incorporation and stability of the CNPs impregnated in the fabric. Figure 5 shows the morphology of the fabric surfaces before and after enzymatic treatment, the fabric impregnated with CNPs, and after the five washing cycles.

**Figure 6:**
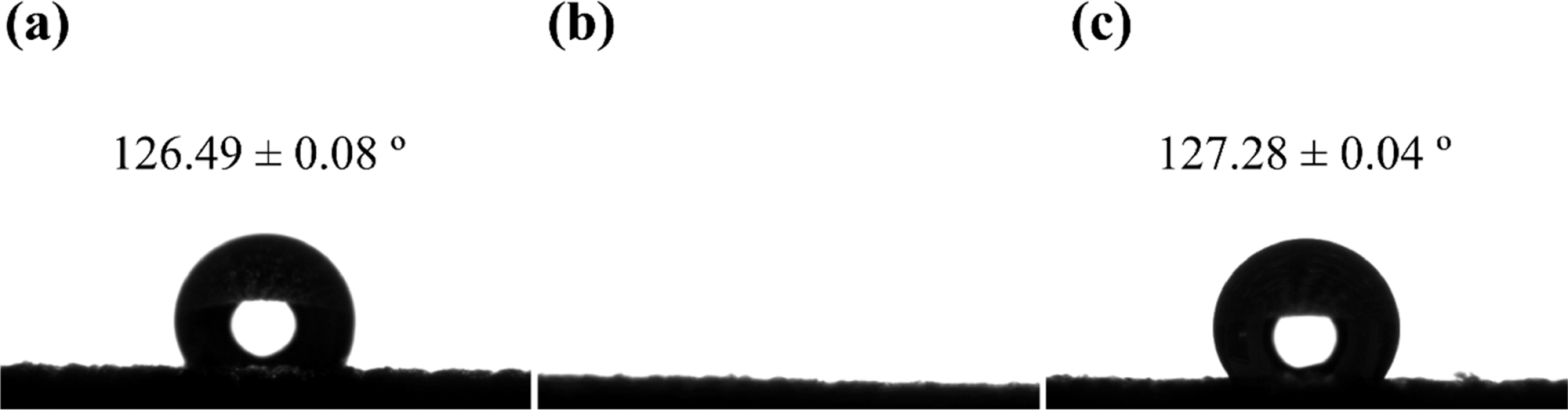
Images of the water drop 0.9 s after being deposited on (a) PA 6 without modifications, (b) *PAHidPP* under optimized conditions, and (c) *PAHidPP_CNPs.*

Unmodified PA 6 shows residual polymer and impurities, such as oils (Figure 6a). After enzymatic modification, the residual polymer and impurities were removed, resulting in a smoother fiber surface without any irregularities. The hydrolyzed PA 6.6 fibers’ clean surface confirms the enzymatic treatment’s effectiveness.^1^

After the CNPs are impregnated, small dots can be found on the fabric’s surface. These nanoparticles have reduced size (10.60 ± 3.26 nm), making them difficult to observe under magnification. However, even after five cycles, the nanoparticles remain visible in the material, indicating the durability of the surface treatment. The durability after washing is a limitation usually found in literature, mainly when the incorporation occurs by intermolecular bonds.^25,26^ This result, therefore, indicates that performing the covalent bond leads to a significant advantage in the stability of the final material.

The modified fabric’s hydrophilicity was evaluated by analyzing the shape of the water drop after 0.9 s of contact with the fabric’s surface. Figure 6 shows images of water droplets on fabric surfaces of PA 6 unmodified, hydrolyzed, and CNPs-incorporated.

The water drop images on the fabric’s surface (Figure 6a,b) demonstrate a higher water-absorbing ability in the hydrolyzed fabric, *PAHidPP,* than the unmodified PA 6. PA 6 takes an average of over 0.9 s to fully absorb a drop, with a contact angle of 126.49 ± 0.08°, whereas *PAHidPP* absorbs it instantly, indicating efficient hydrolysis. This modification increases PA 6’s hydrophilicity, evidenced by the presence of amino and carboxyl groups, resulting in a superhydrophilic fabric.

In contrast, *PAHidPP_CNPs* did not absorb the drop instantly, with a contact angle of 127.28 ± 0.04°, suggesting successful incorporation of CNPs. The covalent bonds formed with the groups generated during hydrolysis hinder immediate water absorption. These results confirm the increased hydrophilicity of *PAHidPP* due to the enzymatic modification and the effective incorporation of CNPs into the fabric surface

**Figure 7:**
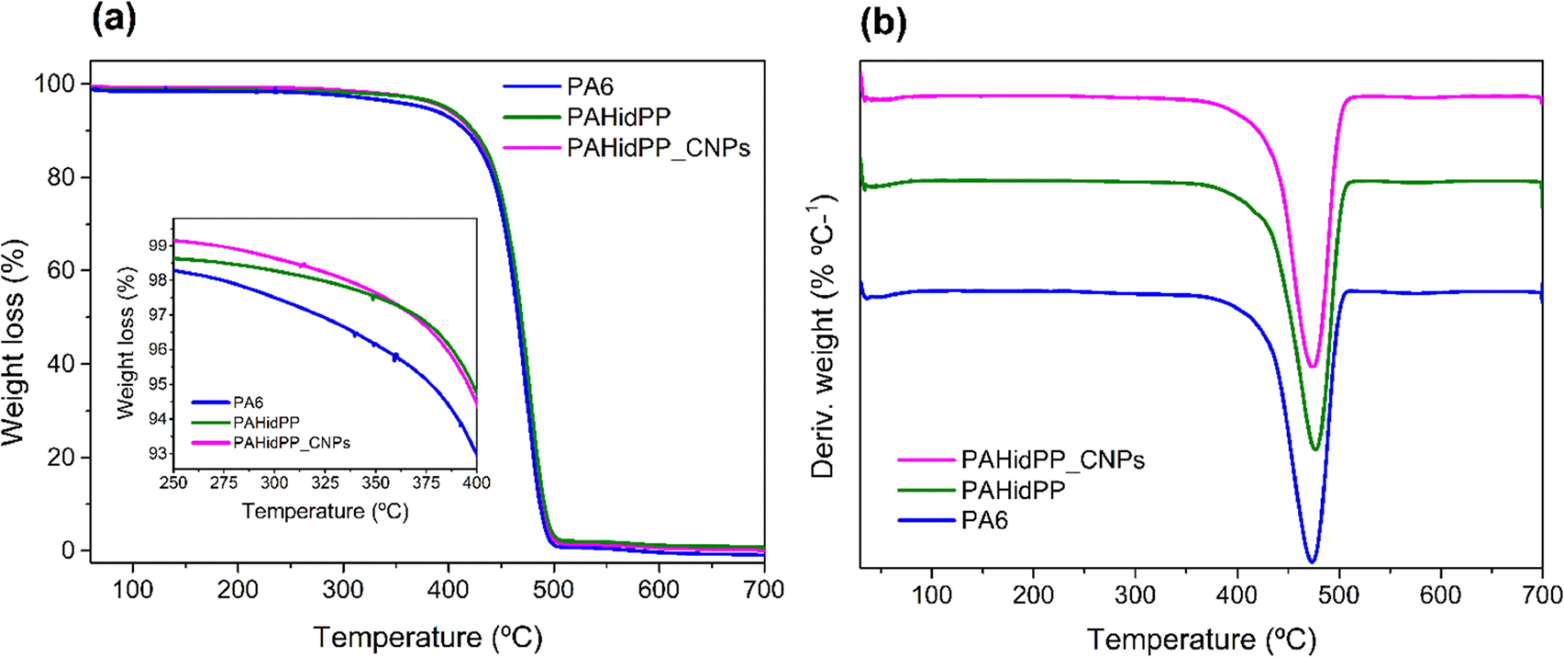
Thermogravimetric analysis of (a) TGA and (b) DTG for PA 6 without modifications, *PAHidPP* under optimized conditions, and *PAHidPP_CNPs*.

The TGA and DTG curves for the PA 6 samples, both unmodified and modified with chitosan nanoparticles, are shown in Figure 7. Table 4 summarizes the initial decomposition temperature (*T*_5%_), determined as the temperature at 5% weight loss, and the decomposition temperature at the maximum degradation rate (*T*_max_), defined through the derivative weight loss curves. Each thermogram displays three distinct stages of weight loss. At temperatures below 100 °C, water and low molecular weight compounds are lost. The second stage, between 370 and 500 °C, results from the thermal decomposition of the polymer chains.^46^ The last stage, from 500 °C onward, corresponds to the thermal decomposition of the polymer chains into ash.^47,48^

**Table 4:** Results of Thermogravimetry Analysis of PA6 without Modifications, *PAHidPP* in the Optimized Conditions, and *PAHidPP_CNPs*

sample	*T*_5%_ (°C)	*T*_max_ (°C)
PA6	377.3	473.7
*PAHidPP*	397.9	477.5
*PAHidPP_CNPs*	395.6	473.1

The TGA curves evidence that PA 6 begins its primary degradation at slightly lower temperatures than the hydrolyzed and modified samples. This initial weight loss might be related to some impurities, as observed in SEM images. The removal of these impurities through the hydrolysis process promoted a higher thermal stability of the fabric. Furthermore, the impregnation of chitosan nanoparticles did not affect this thermal stability. There were no appreciable changes in the *T*_5%_, *T*_max,_ and final residue for *PAHidPP_CNPs,* indicating similar thermal stability to the original material. The obtained result suggests a minimal difference in the decomposition temperature of the modified fabric, maintaining its thermal stability.^47−49^

The bacterial reduction of unmodified PA 6 and *PAHidPP_CNPs* was determined against Gram-positive (*S. aureus*) and Gram-negative (*E. coli*) microorganisms. Table 5 presents the maximum bacterial reduction of both tested bacteria for unmodified and functionalized fabrics.

**Table 5:** Bacterial Reduction Results of *PAHidPP_CNPs* in Comparison to the Control Sample (Non-Functionalized PA)

sample	bacterial reduction (%)	
	*S. aureus*	*E. coli*
*PAHidPP_CNPs*	31.8 ± 9.9	35.6 ± 3.1

The bacterial growth occurred in both samples (PA 6 without modifications and *PAHidPP_CNPs*). Compared to the unmodified fabric, bacterial growth was reduced in *PAHidPP_CNPs*. This increased antibacterial effect of *PAHidPP_CNPs* is related to chitosan’s antimicrobial properties, arising from the interaction between its amino group’s charges and microbial cell walls. This inhibits the growth of both Gram-positive and Gram-negative microorganisms.^40^

The observed results show that the antibacterial activity of the modified fabric reduced the count of *S. aureus* and *E. coli* by approximately 31.8 and 35.6% compared to the untreated fabric, respectively, after 24 h. This reduction in *S. aureus* activity is notably more significant than in a similar study that used CNPs in PA 6 fabric, which reported approximately a 5% reduction for Gram-positive *S. aureus* and Gram-negative *Pseudomonas aeruginosa* when applying CNPs alone. In this study, the reduction was just possible by applying Ag Nps in combination with CNPs.^50^ Compared to the literature on synthetic materials, these results are promising.

**Figure 8:**
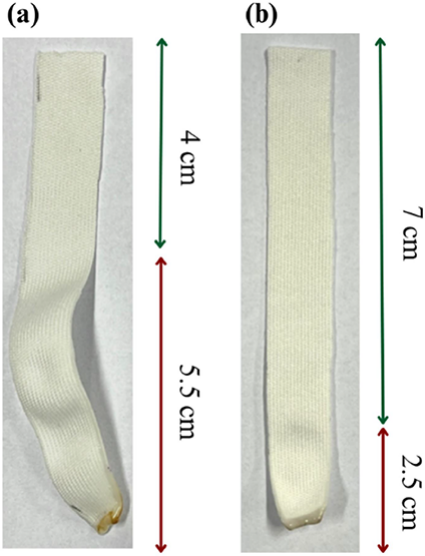
Images of (a) unmodified PA 6 and (b) *PAHidPP_CNPs* after flammability tests.

The chemical structure of chitosan suggests using it as a carbon and nitrogen source for flame retardant formulations. Chitosan can form a stable carbon char if applied to a polymer substrate in a char formation mechanism.^50^ Another possibility is the release of the nitrogen groups present in its structure during the degradation step.^51^ Because of this, functionalized fabric’s effectiveness in improving flame-retardant properties, such as ignition resistance and lateral flame spread, was evaluated using a custom setup adapted from the ASTM D6413-08 flammability test.^52^Figure 8a,b displays images of the unmodified and modified PA 6 samples after the vertical flame test. Detailed results are provided in Table 6.

**Table 6:** Results of the Vertical Flame Test Performed on PA6 without Modifications and *PAHidPP_CNPs*

ASTM D6413 results	PA6	*PAHidPP_CNPs*
initial length (cm)	9.5	9.5
after-flame length (cm)	4.0	7.0
occurrence of melting or dripping	no	no
comments	extinguished instantaneously, the compromised portion became rigid and curved	extinguished instantaneously

Self-extinguishing behavior was observed for unmodified and modified samples without vertical flame spread or material dripping. The unmodified sample shows low resistance to the flame, resulting in 5.5 cm of the sample being compromised after 2 s of exposure to the fire. Although the sample was not degraded and no material dripping was observed, a significant portion lost its original properties, becoming rigid and curved (Figure 7a). In contrast, the CNPs modified sample significantly improved ignition resistance, obtaining reduced damage to the material, compromising only 2.5 cm of the sample, as seen in Figure 7b.

When comparing the portions of the fabrics that remained intact after exposure to flame, it was observed that *PAHidPP_CNPs* preserved 1.75 times more material than the untreated fabric. This result suggests that incorporating chitosan nanoparticles protected against the spread of combustion, giving the functionalized fabric improved flame-retardant properties.^53−55^

In a study by Kundu et al., it was only possible to improve the flame-retardant properties of PA 6 by combining CNPs with other nanoparticles, such as TiO_2_, SiO_2_, and hypophosphite. Therefore, the findings of our study, which may be related to the greater efficiency of covalent bond impregnation on the fabric surface, should be highlighted as a promising alternative to confer this improved property to PA 6 fabric.^47,48^

**Figure 9:**
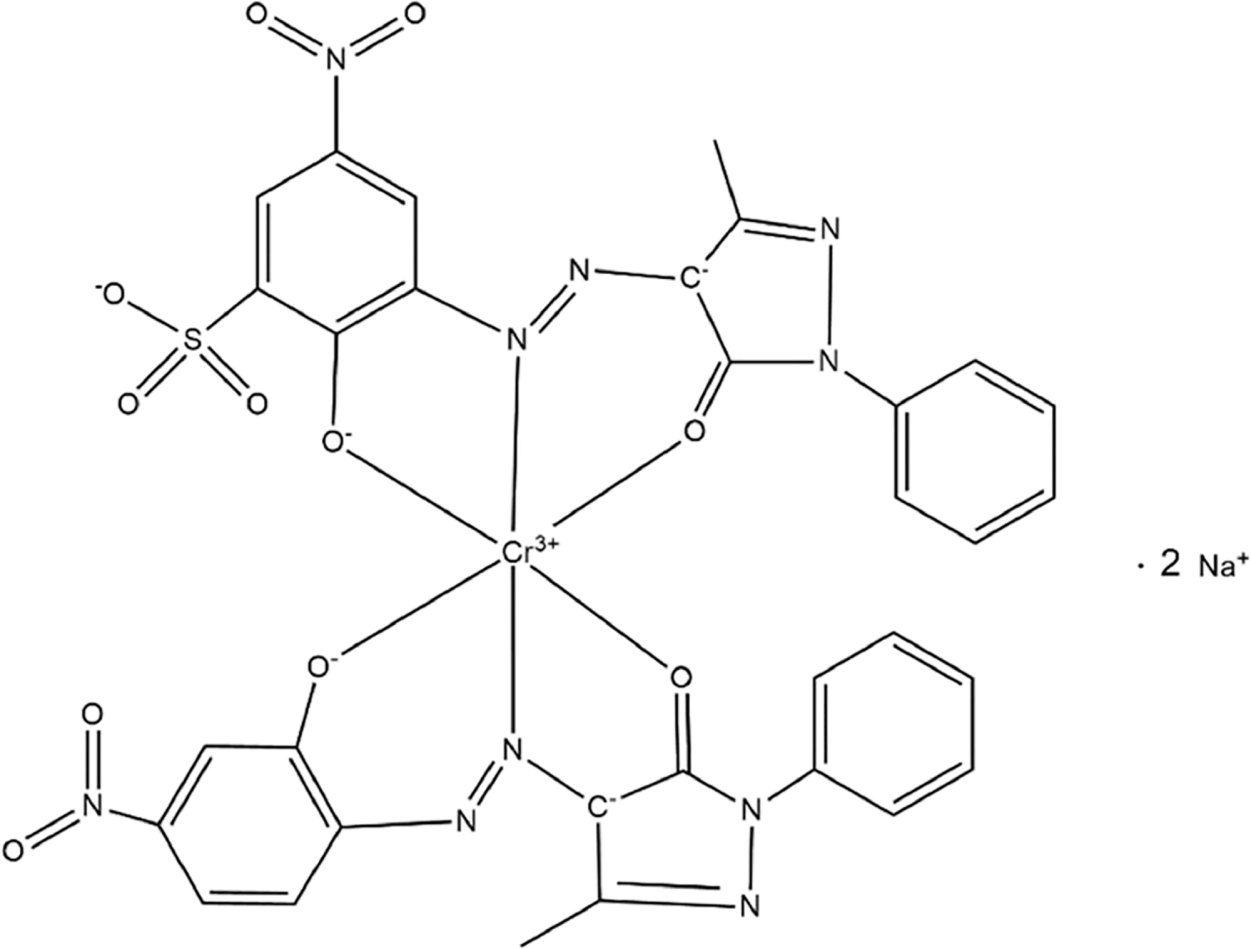
Bemaplex Red MT structure.

The dyeing process was performed for the unmodified and hydrolyzed fabric, which was incorporated with CNPs. After five washing cycles, the fabric incorporated with CNPs was also submitted to the dyeing process. The aim was to confirm and validate the dyeing performance and washing fastness. Table 7 shows the K/S values for each sample after dyeing with the Bemaplex Red MT acid dye (Figure 9) and the fastness values on PA 6 and CO fabrics.

**Table 7:** Washing Fastness Values for ABNT NBR ISO 105: Color Fastness Tests Performed on PA 6 without Modification, *PAHidPP_CNPs,* and 5WC*PAHidPP_CNPs* Dyed with Bemaplex Red MT Acid Dye

sample^a^	*K*/*S*	staining on PA6	staining on CO
PA6	21.629	5	5
*PAHidPP*	22.967	5	5
*PAHidPP_CNPs*	22.083	5	5
5WC*PAHidPP_CNPs*	22.029	5	5

a Dyeing conditions: 1.0% dye (w/w), 0.5 (v/v) acid donor, 95 °C, 60 min.

The wash fastness tests showed excellent results for all the samples, with the maximum rating (5). The dye used has a high affinity for PA 6 fabrics, providing optimum dyeing performance on the unmodified fabric.^13^ However, after enzymatic hydrolysis, the dye’s adsorption increased, resulting in a deeper color, as the higher *K*/*S* values indicated. This increase is attributed to the formation of NH_2_ groups during enzymatic hydrolysis, which increases the dye’s hydrophilicity and affinity.^56^

The incorporation of CNPs maintained this improved performance compared to the unmodified fabric, indicating that surface modification can slightly improve the dyeing properties of PA 6. This performance was maintained even after five washing cycles, suggesting the modification’s durability.

## Conclusions

The surface hydrolysis of PA 6 was carried out using 14 different enzymes, with the lipase from *Porcine pancreas* type II (lyophilized) showing the most favorable results. The optimization of the modification process was effective, leading to an optimum condition with an enzyme concentration of 5%, a reaction time of 90 min, a pH of 8.0, and a temperature of 40 °C. Under these conditions, there was a significant increase in the efficiency of the hydrolysis measured by the *K*/*S* value and by the concentration of MB in the sample (20.5 and 24.8 times higher, respectively, compared to the unmodified fabric). The optimized methodology provided a mild but effective process. *PAHidPP* exhibited increased hydrophilicity due to carboxyl and amino groups forming on the surface, facilitating the incorporation of chitosan nanoparticles through covalent bonds. Modifying the fabric resulted in a smoother surface and improved hydrolyzing capacity, which contributed to more efficient moisture transport in the fabric, optimizing its dyeing properties.

The CNP impregnation on the *PAHidPP* surface was performed using a mild and durable methodology. The treated fabric exhibited a reduction of microbial proliferation against both *S. aureus* and *E. coli*, decreasing their count by approximately 31.8 and 35.6%, respectively, after 24 h. A superior flame-retardant capacity was evaluated, with flame retardancy 1.75 times greater than the unmodified fabric’s. In addition, SEM images and a dyeing fastness assay performed after 5 washing cycles show excellent durability of the modified fabric.

This modification method shows significant potential for industrial application. It represents an ecological process that reduces adverse environmental impacts and requires less energy, water, and time. In addition to improving the beneficiation process, the method also contributes to creating high-performance, value-added material.

## Abbreviations

PA: polyamide
PA6: polyamide 6
CNPs: chitosan nanoparticles.
